# Contact-Induced Mitochondrial Polarization Supports HIV-1 Virological Synapse Formation

**DOI:** 10.1128/JVI.02425-14

**Published:** 2014-12-16

**Authors:** Elisabetta Groppelli, Shimona Starling, Clare Jolly

**Affiliations:** Division of Infection and Immunity, University College London, London, United Kingdom

## Abstract

Rapid HIV-1 spread between CD4 T lymphocytes occurs at retrovirus-induced immune cell contacts called virological synapses (VS). VS are associated with striking T cell polarization and localized virus budding at the site of contact that facilitates cell-cell spread. In addition to this, spatial clustering of organelles, including mitochondria, to the contact zone has been previously shown. However, whether cell-cell contact specifically induces dynamic T cell remodeling during VS formation and what regulates this process remain unclear. Here, we report that contact between an HIV-1-infected T cell and an uninfected target T cell specifically triggers polarization of mitochondria concomitant with recruitment of the major HIV-1 structural protein Gag to the site of cell-cell contact. Using fixed and live-cell imaging, we show that mitochondrial and Gag polarization in HIV-1-infected T cells occurs within minutes of contact with target T cells, requires the formation of stable cell-cell contacts, and is an active, calcium-dependent process. We also find that perturbation of mitochondrial polarization impairs cell-cell spread of HIV-1 at the VS. Taken together, these data suggest that HIV-1-infected T cells are able to sense and respond to contact with susceptible target cells and undergo dynamic cytoplasmic remodeling to create a synaptic environment that supports efficient HIV-1 VS formation between CD4 T lymphocytes.

**IMPORTANCE** HIV-1 remains one of the major global health challenges of modern times. The capacity of HIV-1 to cause disease depends on the virus's ability to spread between immune cells, most notably CD4 T lymphocytes. Cell-cell transmission is the most efficient way of HIV-1 spread and occurs at the virological synapse (VS). The VS forms at the site of contact between an infected cell and an uninfected cell and is characterized by polarized assembly and budding of virions and clustering of cellular organelles, including mitochondria. Here, we show that cell-cell contact induces rapid recruitment of mitochondria to the contact site and that this supports efficient VS formation and consequently cell-cell spread. Additionally, we observed that cell-cell contact induces a mitochondrion-dependent increase in intracellular calcium, indicative of cellular signaling. Taken together, our data suggest that VS formation is a regulated process and thus a potential target to block HIV-1 cell-cell spread.

## INTRODUCTION

Human immunodeficiency virus type 1 (HIV-1) can disseminate between susceptible target T cells via two mechanisms: cell-free infection and direct cell-cell spread. Cell-to-cell spread of HIV-1 occurs across specialized immune cell contacts called virological synapses (VS)—dynamic but transient intercellular junctions at which viral proteins, entry receptors, and adhesion molecules are concentrated (1, 2). The local accumulation of viral proteins at the VS demarks them as sites of preferential HIV-1 assembly and egress, resulting in polarized budding of virus into the synaptic cleft and leading to rapid infection of the target cell that is in close physical contact (1, 3–7). Indeed, it has been estimated that cell-cell spread of HIV-1 between T cells is approximately 1 order of magnitude more efficient than equivalent cell-free infection that is dependent on fluid-phase diffusion (2–4, 7–10). In addition, the increased local concentration of virus and limited time exposed to the external milieu may provide a means to avoid inhibition by antiviral antagonists, including neutralizing antibodies, cellular restriction factors, and some components of antiretroviral therapy (5, 11–18). The replicative advantage of cell-cell spread at VS may be particularly important in lymphoid tissue, where CD4 T cells are densely packed and likely to frequently interact, and recent intravital imaging studies have validated the concept of the VS *in vivo* (19, 20). Thus, cell-cell spread is likely to play an important role in HIV-1 replication and pathogenesis and presents a formidable barrier to eradication of the virus from the host.

Immune cells such as T cells are not inherently polarized and do not show strong front-rear polarity in the absence of stimulation; hence, organelles are usually evenly distributed within the cytosol. However, T cells can adopt front-rear polarity following stimulation through cell-cell contact with antigen-presenting cells (APC) at the immunological synapse (IS) (21–24) and during migration and in response to soluble stimuli such as chemokines (25). During IS formation, contact with an APC and subsequent T cell receptor (TCR)-induced signaling trigger rapid cytoplasmic and membrane remodeling within the T cell that recruits organelles such as mitochondria, the secretory apparatus, and signaling machinery to the contact site (26). Mitochondria play a particularly important role at the IS by supporting sustained calcium influx that is required to support synaptic signaling, IS formation, and T cell effector functions (22, 23, 25, 27). The HIV-1 VS shares many similarities with the IS (28, 29), including synaptic enrichment of adhesion proteins such as lymphocyte function-associated antigen 1 (LFA-1)/intercellular adhesion molecule (ICAM), formation of a receptor-bearing complex at the contact zone, cytoskeletal rearrangements, and polarized budding of virus that is reminiscent of polarized secretion from T cells at the IS. An important difference is that while the IS is driven by TCR–peptide-major histocompatibility complex (pMHC) binding, the HIV-1 T cell VS is TCR independent and instead requires Env-CD4 and adhesion protein interactions (30).

Intriguingly, recent electron microscopy (EM) tomographic reconstructions of the HIV-1 T cell VS have revealed striking cytoplasmic polarization and clustering of organelles (e.g., mitochondria and the microtubule organizing center [MTOC]) proximal to the plasma membrane at the contact zone that were spatially associated with HIV-1 budding (31). Although this polarization was not seen in HIV-1-infected cells in the absence of cell-cell contact, it remained unclear whether T cell polarization at the VS was triggered by cell-cell contact or, rather, if contacts were formed only by a proportion of cells with some preassembled polarized phenotype. Therefore, whether HIV-1-infected T cells can sense and respond to contact to create a localized environment that supports efficient cell-cell spread is unresolved, and the precise mechanisms leading to VS formation and how this is regulated remain to be elucidated.

Here, we have coupled fixed and live-cell imaging with functional virology and show that HIV-1-infected T cells rapidly remodel in response to contact with target T cells and that this is an active and calcium-dependent process culminating in the polarization of mitochondria and HIV-1 Gag to the site of cell-cell contact. Polarization occurred within minutes of cell-cell contact, required the formation of a stable contact, and displayed kinetics similar to the IS. Further analysis revealed that a transient rise in cytoplasmic calcium concentration is coupled with VS formation and mitochondrial polarization, further supporting the hypothesis that organelle polarization at the VS is a regulated process. We conclude that HIV-1-infected cells can rapidly sense cell-cell contact and remodel their shape to adopt a polarized phenotype. This represents a mechanism by which T cells can create a localized synaptic environment supporting VS formation and efficient HIV-1 transmission between CD4 T lymphocytes.

## MATERIALS AND METHODS

### Cells, viruses, and tissue culture.

Jurkat CE6.1 and the derivative Jurkat line 1G5 (from Estuardo Aguilar-Cordova and John Belmont) containing the firefly luciferase gene driven by the HIV-1 long terminal repeat (LTR) were obtained through the AIDS Research and Reference Reagent Program (ARRRP), Division of AIDS, NIAID. LFA-1-negative Jurkat (β2.7) and parental LFA-1-expressing (Jn9) cells were from L. Klickstein (Harvard University, Cambridge, MA, USA) (32, 33). CD4^−^ T cells (A2.01) and parental CD4 T cells (A3.01) were from the Center for AIDS Reagents (CFAR), National Institute for Biological Standards and Control (NIBSC), United Kingdom. Primary CD4 T cells were isolated from peripheral blood mononuclear cells by Ficoll-Hypaque gradient and negative selection (Miltenyi Biotec) and maintained as described previously (1). The HIV-1 clone pNL4.3 generated by Malcolm Martin was from the ARRRP. The derivative pNL4.3-green fluorescent protein (GFP) was a kind gift from Barbara Muller (34). Infectious virus was produced by transfecting pNL4.3 plasmid or cotransfecting pNL4.3-GFP with wild-type (WT) HIV-1 Gag into 293T cells using Fugene 6 (Promega). Virus was harvested after 48 h, and infectious titer was measured on HeLa TZM-bl reporter cells (donated by J. Kappes, X. Wu, and Tranzyme Inc. and obtained from the CFAR, NIBSC, United Kingdom) using the Bright-Glo luciferase assay kit (Promega). Jurkat and primary CD4 T cells were infected by spinoculation at 1,200 × *g* for 2 h at a multiplicity of infection (MOI) of 0.1 to 0.2. HIV-1 infection was monitored by flow cytometry of intracellular Gag and surface Env as described previously (31). Cells were used 48 to 72 h postspinoculation when >90% of the cells were HIV-1 infected.

### Immunofluorescence (IF) microscopy.

HIV-1-infected T cells were washed, mixed with an equal number of uninfected target T cells stained with CellTrace FarRed (Life Technologies), and incubated on poly-l-lysine (Sigma)-treated coverslips at 37°C for up to 60 min. At various times postmixing, the cells were fixed in 4% formaldehyde in phosphate-buffered saline (PBS)–1% bovine serum albumin (BSA) for 15 min, washed, and permeabilized either in 0.1% Triton X-100–5% fetal calf serum (FCS) for 20 min at room temperature (RT) or in 100% cold methanol for 5 min. Cells were stained using the following antibodies: HIV-1 Gag with rabbit antisera against Gag p17 and p24 (donated by G. Reid and obtained from the CFAR, NIBSC, United Kingdom), mitochondria with anti-ATPβ (Abcam), HIV-1 Env with the nonblocking 50-69 (CFAR), and CD4 with the nonblocking monoclonal antibody (MAb) L120 (CFAR). 50-69 and L120 were included during VS formation (1). Primary antibodies were detected with either fluorescein isothiocyanate (FITC), tetramethyl rhodamine isocyanate (TRITC), Cy5 (Jackson ImmunoResearch), or Alexa-conjugated (Life Technologies) anti-mouse or -rabbit secondary antibodies that were tested for an absence of interspecies reactivity. Coverslips were mounted with ProLong antifade mounting solution (Life Technologies), and fixed cells were imaged using either a Leica SP2 laser scanning confocal microscope or a DeltaVision Elite Image Restoration microscope (Applied Precision/Olympus) through a 60× 1.4-numerical-aperture (NA) oil immersion lens with an inverted Olympus IX71 microscope and a CoolSNAP HQ2 or Evolve camera. Images were acquired through the entire volume of the cell and deconvolved with SoftWoRx 5.0. Processing was performed using Huygens Professional version 4.0, MetaMorph version 7.1, and Adobe Photoshop CS6. Integrated fluorescence intensity analysis of cell-cell contacts was performed using MetaMorph. Briefly, differential interference contrast (DIC) images were used to segment the infected cell into 3 rectangular regions of equal size starting from the site of contact with the uninfected cell, identifying the proximal, medial, and distal thirds of the cell. The 3 regions were then automatically applied to the corresponding fluorescent images, and the integrated fluorescence intensity of individual fluorescent channels was recorded and expressed as a percentage.

### Live-cell imaging.

For live-cell imaging, Jurkat or primary CD4 T cells were infected with NL4.3-GFP for 48 to 72 h, washed, stained with 250 nM MitoTracker (Life Technologies), and resuspended in RPMI medium without phenol red. Uninfected target T cells were either left untreated or stained with CellTrace FarRed (Life Technologies). Equal numbers of infected and uninfected cells were mixed in a poly-l-lysine-coated chambered cover glass (LabTek chambered borosilicate cover glass) and imaged immediately at 37°C, 5% CO_2_ (DeltaVision Elite Image Restoration microscope). Time-lapse recordings were made every 2 min for a total of 60 min from randomly selected fields. Acquisition was performed with Real-Time-Z-Sweep in which the Z dimension is acquired continuously and automatically converted to maximum intensity projection. Images were deconvolved using SoftWoRx 5.0 and processed using Huygens Professional version 4.0, MetaMorph version 7.1, and Adobe Photoshop CS6. For motility assays, infected cells pretreated or not with Mdivi were loaded with CellTrace Green (Life Technologies) and imaged every 2 min for a total of 46 min at 37°C, 5% CO_2_ (DeltaVision Elite Image Restoration Microscope). Data analysis was performed with Huygens Professional version 4.0.

### Quantification of HIV-1 T cell-T cell spread and cell-free virus production.

HIV-1-infected T cells were washed, mixed with an equal number of 1G5 Jurkat T cells, and incubated for 18 h at 37°C. 1G5 Jurkat cells contain the firefly luciferase gene driven by the HIV-1 LTR. Luciferase activity was measured by luminescence assay using the Bright-Glo luciferase assay (Promega). For cell-free virus production, HIV-1-infected cells were incubated without targets, the virus-containing supernatants were harvested, and p24 levels were measured with an enzyme-linked immunosorbent assay (ELISA) (35).

### RNA interference (RNAi) knockdown of Drp1 in T cells.

Jurkat T cells were transfected using Nucleofection (nucleofected) (Amaxa; Lonza) with 5 μM small interfering RNA (siRNA) oligonucleotides targeting Drp1 or nontargeting control sequences (Dharmacon Smartpool; Thermo), and 24 h later, the cells were infected with pNL4.3 virus by spinoculation. Knockdown of Drp1 was confirmed by SDS-PAGE and Western blotting at 72 h postnucleofection using MAbs against Drp1 (BD Bioscience) and glyceraldehyde-3-phosphate dehydrogenase (GAPDH) (Abcam) as a loading control and visualized by enhanced chemiluminescence (ECL) (GE Healthcare). Quantification was performed using Image J.

### Pharmacological inhibitors.

HIV-1-infected T cells were preincubated with 100 μM 2-aminoethyl diphenylborate (2-APB) (Merck), 5 μM oligomycin (Sigma), 1 μM nocodazole (Sigma), 50 μM Mdivi (Sigma), 10 mM EGTA (Fluka), 10 μM BAPTA-AM (Life Technologies), or 0.5% dimethyl sulfoxide (DMSO) as a control for 30 min prior to mixing with target T cells. 2-APB was removed by washing after treatment of HIV-1-infected cells. Infected cells were then mixed with target cells and processed for immunofluorescence microscopy as described above. Cell-free virus production was quantified with an enzyme-linked immunosorbent assay (ELISA) to measure p24 (35). Viability and ATP concentration of the treated cells were measured by trypan blue exclusion (Life Technologies) and CellTiter-Glo (Promega), respectively.

### ImageStream.

Infected T cells were loaded concomitantly with Fluo4-AM calcium indicator (Life Technologies) and CellTrace Violet (Life Technologies), while uninfected target cells were stained with CellTrace FarRed (Life Technologies). Equal numbers of infected and uninfected cells were mixed and either fixed immediately in ice-cold 4% formaldehyde or allowed to interact for 10, 20, and 40 min before fixing. Samples of uninfected cells treated in the same way as infected cells (i.e., loaded with Fluo4 and Violet) were included in the panel as target-target control. After fixing, the cells were gently washed in PBS and then analyzed with ImageStream (Amnis, EMD Millipore). Acquisition gating for single cells or doublets was determined by plotting aspect ratio intensity versus area, and the gate boundaries were optimized by visual inspection of random events. Fluorescent channel compensation and data analysis were performed with IDEAS software (Amnis, EMD Millipore). Briefly, acquired doublets were enriched for events in focus (gradient RMS) and positive for both CellTrace Violet (infected cell) and CellTrace FarRed (uninfected cell). As for acquisition gating, the gate boundaries were stringently determined by visual inspection of random events. The mean fluorescence intensity of Fluo4 was then quantified. To determine the Fluo4 signal of single cells (uninfected or infected), cells were loaded with CellTrace Violet and Fluo4 and fixed and processed as described above with gating optimized for single cells.

### Statistical analysis.

Statistical significance was calculated for normally distributed data using the parametric analysis of variance (ANOVA) for multiple comparisons with the Bonferroni correction. The frequencies of mitochondrial polarization were compared by using the odds ratio (OR), and *P* was calculated using Fisher's exact test for nonnormally distributed data. Significance was assumed when *P* was <0.05.

## RESULTS

### HIV-1-infected T cells polarize in response to cell-cell contact.

**FIG 1:**
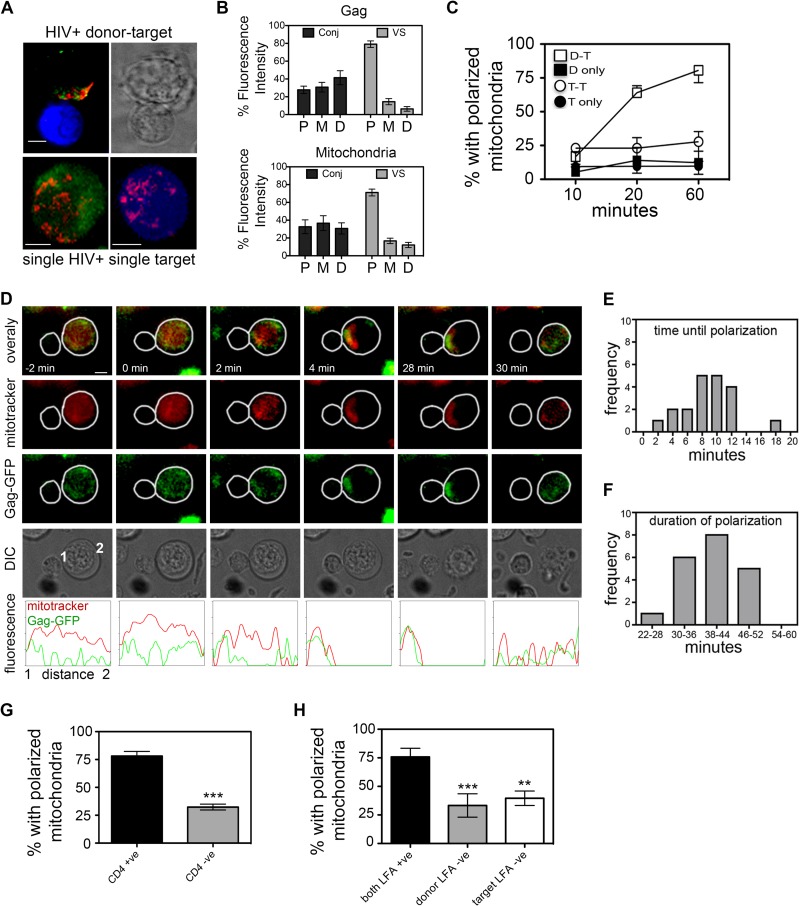
Mitochondria are actively recruited in HIV-1-infected T cells to sites of T cell contact. (A) Representative images of VS and single T cells. Green, Gag; red, mitochondria; blue, target cell (see Movie S1 in the supplemental material). Bar, 5 μm. (B) Automated integrated fluorescence intensity analysis of Gag (top panel) and mitochondria (bottom panel) in the interface proximal (P), medial (M), and distal (D) thirds of an infected cell engaged in a contact with a target cell was performed using MetaMorph. Gag and mitochondria were defined as polarized when >70% fluorescence intensity was detected in the proximal third. The integrated fluorescence intensity data are shown for cells that have formed a VS (*n* = 25) and for infected and uninfected contacts (conjugate, *n* = 25) that have not formed a VS. Copolarization to the VS is evident in three-dimensional reconstructions of fluorescence images (see Movie S1 in the supplemental material). (C) Time course of the percentage of cells with mitochondria polarized to contact site or single cells showing mitochondria polarized. VS, contacts between HIV^+^ cells and target T cells (*n* = 149 cells); TT, contacts between two uninfected target cells (*n* = 125 cells); D, unconjugated HIV^+^ cell (*n* = 137 cells); T, unconjugated target cell (*n* = 141 cells). (D) HIV-1-infected Jurkat cells (Gag-GFP) loaded with MitoTracker dye (red) were mixed with uninfected primary CD4 T cells (unlabeled) and imaged by time-lapse microscopy (*n* = >350 HIV-1-infected Jurkat cells imaged). Representative images extracted from time-lapse (see Movie S3 in the supplemental material) initiation of cell-cell contact at *t* = 0 min. Line scan fluorescence intensity profiles are across the HIV-1-infected T cell from contact interface (point 1) to distal edge (point 2). (E and F) Kinetics of mitochondrial polarization in HIV-1-infected T cells during cell-cell contact from live-cell imaging of Jurkat cells (*n* = 20 VS). Frequency distribution of polarization time (E) and duration of polarization (F). Initiation of cell-cell contact, *t* = 0 min. (G) Percentage of HIV-1-infected cells polarizing during contact with uninfected CD4^+^ A3.01 or CD4^−^ A2.01 target T cells (*n* = >74 contacts per condition). (H) Requirement for LFA-1–ICAM during contact-induced polarization. Black bars, HIV-1-infected LFA-1-positive T cells and LFA-1-positive targets; gray bars, HIV-1-infected LFA-1-negative T cells and LFA-1-positive targets (no LFA-1 engaged on HIV-1^+^ cell); white bars, HIV-1-infected LFA-1-positive cells and LFA-1-negative targets (no ICAM-1 engaged on HIV^+^ cell) (*n* = >33 contacts per condition). For all data: **, *P* < 0.01; ***, *P* < 0.001 (three independent experiments).

To investigate whether T cell polarization at the VS can be triggered by T cell-T cell contact, immunofluorescence microscopy was performed to examine the cellular distribution of mitochondria and HIV-1 Gag in infected T cells in the presence and absence of cell-cell contact. We have previously shown that the VS is associated with polarized clustering of cellular organelles, including mitochondria, in the HIV-1-infected T cell at the site of cell-cell contact (31). Because mitochondria play a key role at immune cell contacts, we focused here on this organelle, with the additional advantage that the dynamics of mitochondrial trafficking to immune cell contacts are relatively well studied and mitochondria can be readily labeled with dyes that are amenable to live-cell imaging. Jurkat T cells were infected with HIV-1, and flow cytometry analysis confirmed that >90% of the cells were Gag^+^ by 48 h postinfection (31). Unless otherwise stated, target cells were always primary CD4 T lymphocytes. HIV-1-infected Jurkat CD4 T cells were mixed in a 1:1 ratio with uninfected primary CD4 T cells and allowed to interact for various periods of time at 37°C before being arrested by formaldehyde fixation, stained with antibodies, and quantified by immunofluorescence (IF) microscopy. The HIV-1 VS is defined by enrichment of viral proteins, such as the capsid protein Gag at intercellular junctions; therefore, localization of HIV-1 Gag acts as a marker of the VS (1, 3, 6). Doublets formed of one HIV-1-infected and one uninfected T cell that showed polarization of Gag to the contact site were identified as VS (1, 4, 6, 7). Polarization was defined as >70% total Gag fluorescence located within the third of the infected cell proximal to the contact site, with the remaining fluorescence distributed in the medial and distal thirds of the cell (Fig. 1A and B). Doublets that did not meet this definition and showed even distribution of Gag among the proximal, medial, and distal thirds of the cell were termed conjugates (Fig. 1B). Once VS and conjugates were identified, the intracellular distribution of antibody-labeled mitochondria was examined. VS were scored as “polarized” if more than 70% of the immunolabeled mitochondria within the cell were clustered at the contact zone or “nonpolarized” if mitochondria were evenly dispersed (Fig. 1A and B). Using this definition of polarization, we found a strong association between Gag enrichment at the contact site (i.e., VS formation) and copolarization of mitochondria within the HIV-1-infected cell (Fig. 1A and B). The coclustering of Gag and mitochondria at the VS is especially evident in volume rendering of IF microscopy sections (see Movie S1 in the supplemental material). Notably, mitochondrial polarization was seen only in the HIV-1-infected T cell, not in the uninfected target T cell coengaged in the VS (Fig. 1A and C). Furthermore, polarization was only infrequently observed when two uninfected target cells were in contact, suggesting a requirement for HIV-1 infection for polarization to occur. This was supported by statistical analysis that revealed that the frequency of polarization in HIV-1-infected T cells was highly significant compared to contacts formed between two uninfected T cells (odds ratio [OR] at 10 min, 1; OR at 20 min, 9; OR at 60 min, 17.9; Fisher's exact test, *P* < 0.0001) (Fig. 1C). Importantly, mitochondria were not polarized in HIV-1-infected T cells that were not in physical contact with another T cell, demonstrating that virus infection alone does not induce cytoplasmic polarization in the absence of cell-cell contact.

The proportion of contact interfaces in which Gag and mitochondria copolarized to the contact site would be expected to remain relatively constant over time if HIV-1-infected T cells were already prepolarized at the moment in which the cells come into contact. Although we did not observe evidence of HIV-1-infected T cells showing a prepolarized phenotype, a time course analysis was performed to specifically test this. We found that the percentage of HIV-1-infected T cells with mitochondria clustered at the contact zone increased during extended coincubation of cells at 37°C (Fig. 1C) (14% polarized after 10 min of coincubation, 65% after 20 min, and 78% after 60 min; *P* = 0.001), indicative of contact-induced T cell polarization that is restricted to the HIV-1-infected T cell.

**FIG 2:**
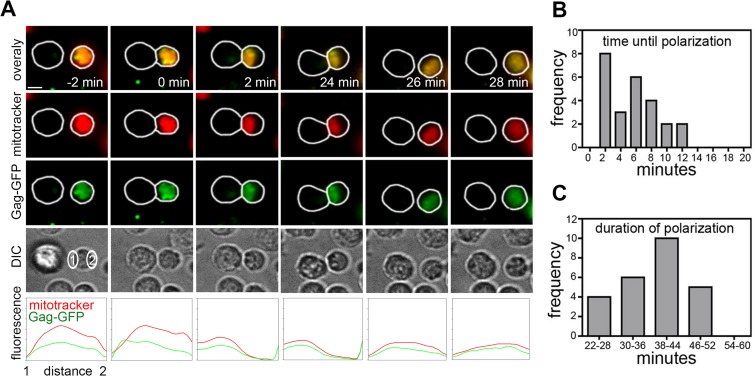
Primary CD4 T cells infected with HIV-1 polarize in response to cell-cell contact. (A) As for Fig. 1D but using HIV-1-infected primary CD4 T cells and autologous uninfected CD4 T cells (>950 HIV-1-infected primary cells imaged from at least 3 independent experiments) (see Movie S4 in the supplemental material). (B and C) Summary of the kinetics of mitochondrial polarization in HIV-1-infected T cells during cell-cell contact from live-cell imaging of primary CD4 T cells (*n* = 25 VS) quantified from 3 independent experiments. Initiation of cell-cell contact, *t* = 0 min.

To analyze the kinetics of polarization in HIV-1-infected cells, live-cell imaging was performed to follow T cell polarization from the moment in which two cells came into contact. Jurkat T cells (Fig. 1) or primary CD4 T cells (Fig. 2) infected *in vitro* with replication-competent, green fluorescent protein-positive (GFP^+^) reporter virus (HIV-1-GFP) were labeled with MitoTracker dye (red), washed, and mixed with uninfected primary CD4 target T cells. The HIV-1-GFP virus has GFP fused to the open reading frame of the major structural protein Gag (34), resulting in a replication-competent virus. In this context, GFP recruitment to sites of cell-cell contact acts as a marker of VS formation (6, 36). In the absence of cell-cell contact, mitochondria and Gag-GFP fluorescence were either diffusely distributed within the cytoplasm of HIV-1-infected Jurkat cells or, alternatively, appeared as randomly distributed cytoplasmic puncta, which are both indicative of a nonpolarized phenotype (Fig. 1D; see also Movies S2 and S3 in the supplemental material). Notably, following contact with the uninfected T cell, mitochondria in the HIV-1-infected T cell rapidly translocated to the contact zone concomitantly with Gag recruitment (Fig. 1D; also see Movies S2 and S3 in the supplemental material). Quantification of multiple contacts revealed that mitochondrial recruitment to the contact site took on average <10 min from the moment of initial cell-cell contact (defined as *t* = 0) (Fig. 1E), similar to estimates of the dynamics of mitochondrial translocation to the IS (23, 25, 37). Transient short-lived contacts of <2 min in duration failed to induce mitochondrial or Gag polarization (see Movie S6 in the supplemental material).

Once at the VS, mitochondria were very stable, indicative of cells maintaining a polarized phenotype, and most cell-cell contacts that we imaged remained polarized for 38 to 44 min up to the point of disengagement, after which mitochondria and Gag rapidly depolarized (Fig. 1F; see also Movies S2 and S3 in the supplemental material). These data are consistent with previous estimates of VS longevity (7). Importantly, cytoplasmic polarization did not occur in the absence of Gag-GFP recruitment, demonstrating that polarization is temporally coupled with VS formation (data not shown). In good agreement with previous estimates of the frequency of VS formation between CD4 T cells (1, 6, 7, 38), quantification of live-cell imaging revealed that 42% (standard error of the mean [SEM], ±5%) of contacts between HIV-1-infected Jurkat T cells and uninfected target resulted in GFP recruitment to the contact interface and VS formation.

The HIV-1 T cell VS is devoid of classical TCR-pMHC interactions that induce IS formation. Instead, it is driven by the engagement of HIV-1 Env on the infected cell with CD4 on the target, with an additional requirement for LFA-1–ICAM interactions (1, 2, 38). Next, we quantified polarization at sites of cell-cell contact using mutant T cell lines in which key receptor interactions known to mediate VS formation were bypassed. We, and others, have shown a requirement for Env for efficient VS formation (1, 3, 4, 19, 20, 38); therefore, to determine whether Env was required for contact-induced polarization, CD4-negative T cells were used as targets and mixed with HIV-1-infected donors, thereby obviating Env engagement on the infected T cell. Figure 1G shows that in the absence of CD4 on target T cells, there was a significant reduction in the frequency of cell-cell contacts showing polarization toward intercellular junctions compared to the CD4-positive parental cell line (32% polarized compared to 78%; *P* < 0.0001, OR = 7.7). Likewise, bypassing cell surface integrin interactions using LFA-1-deficient Jurkat cells (32) as HIV-1-infected donors also significantly reduced the percentage of contacts showing mitochondrial polarization (32% with LFA-1-negative cells compared to 80% with LFA-1-positive cells; *P* < 0.0001, OR = 7.1) (Fig. 1H). Bypassing ICAM-1 engagement on the HIV-1-infected T cell by using LFA-1-deficient cells as targets also reduced polarization (43% polarization; *P* = 0.003, OR = 4.4) (Fig. 1H). Taken together, these data confirm that CD4-Env and LFA-1–ICAM interactions are necessary for contact-induced cytoplasmic remodeling in HIV-1-infected T cells. This is supportive of our live-cell imaging in which transient and unstable contacts of <2 min in duration failed to polarize.

### Primary CD4 T cells infected with HIV-1 polarize in response to cell-cell contact.

Next, we performed live-cell imaging of HIV-1-infected primary CD4 T cells mixed with autologous uninfected CD4 T cells. Similar but slightly faster kinetics of contact-induced polarization to the VS were observed compared to Jurkat cells (Fig. 2A, B, and C; see also Movies S4 and S5 in the supplemental material). As in the case of Jurkat cells, mitochondria were seen to translocate to the synapse alongside Gag-GFP and redistribute within the cytoplasm upon termination of contact (data not shown), suggesting that establishment of cell-cell contact, polarization, and VS formation are tightly linked. Consistent with data obtained using Jurkat cells, a similar proportion of contacts between HIV-1-infected primary T cells and autologous uninfected target cells formed VS (39%; SEM, ±8%). Taken together, these data demonstrate that T cell remodeling and polarization occur in response to cell-cell contact and also confirm Jurkat T cells as a relevant model system in which to interrogate contact-induced polarization at VS.

### Contact-induced mitochondrial polarization is active and calcium dependent.

**FIG 3:**
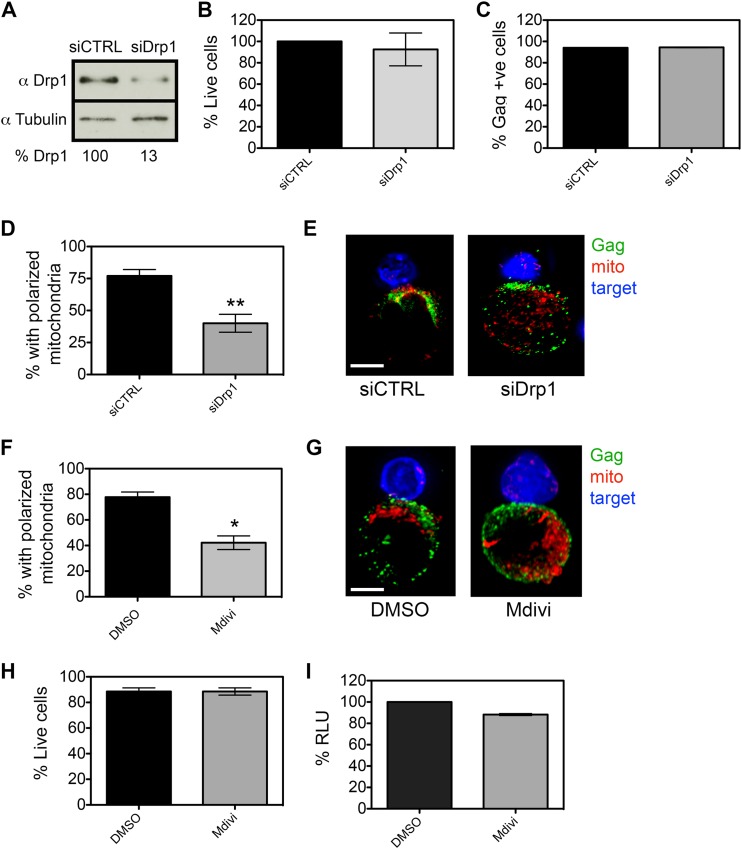
Contact-induced mitochondrial polarization is an active process. (A) Western blot at 72 h after nucleofection of siRNA targeting Drp1 (or control siRNA) in Jurkat T cells. (B and C) Percent viability (% live cells, Drp1 versus control) and percent infection (% Gag-positive cells) for cells nucleofected with siRNA targeting Drp1 or control siRNA. (D) siRNA-nucleofected cells were infected with HIV-1, and mitochondrial polarization at the VS was quantified. siRNA knockdown of Drp1 reduced mitochondrial polarization in HIV-1-infected T cells to the contact zone by 35% (**, *P* < 0.01). (E) Representative image from panel D (*n* = 30). Red, mitochondria; green, Gag; blue, target cell. Bar, 5 μm. (F) Treating HIV-1-infected cells with Mdivi before mixing with uninfected target cells reduced the number of contacts showing mitochondrial polarization by 43% (*, *P* < 0.05). (G) Representative image from panel F (*n* = 30). Red, mitochondria; green, HIV-1 Gag; blue, target T cell. Bar, 5 μm. Representative images are volume projections extracted from three-dimensional reconstructions (see Movies S7 and S8 in the supplemental material). Note that, as expected, inhibiting Drp1 with Mdivi results in elongated mitochondrial tubules. (H and I) Mdivi treatment does not reduce cell viability (trypan blue exclusion) (H) or ATP production (% relative light units [RLU]) (I).

Mitochondrial trafficking in T cells requires unopposed organelle reshaping via fission mediated by Drp1. Drp1 regulates mitochondrial positioning in T cells by allowing mitochondria to reshape into smaller and more mobile structures (27, 39). We used siRNA to specifically deplete Jurkat T cells of Drp1 and examined the consequences for mitochondrial polarization to the VS. Nucleofection resulted in an 87% decrease in cellular Drp1 protein (Fig. 3A) without compromising cell viability (Fig. 3B). Drp1-depleted cells could be infected similarly to control cells (Fig. 3C) and therefore could be used as donor cells in VS assays. Figures 3D and E show that siRNA knockdown of Drp1 reduced mitochondrial polarization in HIV-1-infected T cells to the contact zone by 35% (*P* < 0.01). As an alternative to siRNA nucleofection, we used Mdivi, a chemical that specifically inhibits Drp1 (40). Treating HIV-1-infected cells with Mdivi before mixing them with uninfected target cells reduced the number of contacts showing mitochondrial polarization by 43% (*P* < 0.05) (Fig. 3F and G) and again had no effect on cell viability and ATP levels (Fig. 3H and I). Failure of Mdivi-treated cells to polarize mitochondria, in comparison to the DMSO control, is especially evident in volume rendering of IF microscopy (see Movies S7 and S8 in the supplemental material).

**FIG 4:**
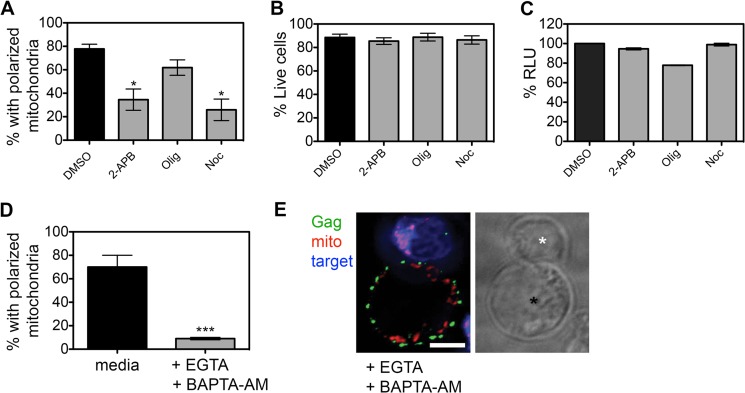
Contact-induced mitochondrial polarization is calcium dependent. (A) HIV-1-infected T cells were treated with 2-APB, nocodazole (Noc), oligomycin (Olig), or DMSO control; mixed with target T cells; stained for HIV-1 Gag and mitochondria; and analyzed by immunofluorescence microscopy. The percentage of contacts showing mitochondrial polarization to the contact interface was quantified. Polarization of mitochondria to the VS is decreased by inhibition of IP_3_R with 2-APB and microtubule depolymerization (Noc) but not by inhibition of ATP synthesis (Olig) (*, *P* < 0.05). (B) Treatment with 2-APB, oligomycin, and nocodazole does not affect cell viability (trypan blue exclusion). (C) Cellular ATP levels are reduced after treatment with oligomycin but not with 2-APB and nocodazole (% relative light units [RLU]). (D) HIV-1-infected T cells were pretreated with EGTA and BAPTA-AM, mixed with target cells, and analyzed by immunofluorescence as described for panel A. Calcium chelation with EGTA and BAPTA-AM reduces mitochondrial polarization to the VS (***, *P* < 0.001). (E) Representative image (*n* = 33) showing Gag (green) and mitochondria (red). HIV-1-infected cells (black asterisk) are single Z sections (0.2 μm). Target cells were labeled with CellTrace FarRed (blue; white asterisk). Bar, 5 μm.

Live-cell imaging revealed that HIV-1-infected T cells rapidly remodeled and polarized within minutes of cell-cell contact, suggestive of active processes (Fig. 1 and 2). In the case of the well-studied IS, polarization of mitochondria is dependent on the initial release of calcium from intracellular stores that is mediated by inositol-1,4,5-triphosphate receptor (IP_3_R). IP_3_R is a tetrameric transmembrane complex found on the endoplasmic reticulum (ER) and Golgi complex that, upon ligand binding (IP_3_), rapidly releases intraluminal Ca^2+^ (41). The pharmacological inhibitor 2-APB has been extensively used to block IP_3_R-mediated calcium release and study T cell polarization at the IS (23, 27, 42, 43). Figure 4A shows that when HIV-1-infected T cells were pretreated with 2-APB, the frequency of VS showing mitochondrial polarization was significantly reduced (53% decrease in polarized conjugates; *P* < 0.01) with no effect on cell viability or ATP levels (Fig. 4B and C). A similar reduction in polarization was also seen when infected T cells were treated with the positive-control nocodazole that inhibits organelle trafficking by depolymerizing microtubules (68% decrease in polarized conjugates; *P* < 0.01) (Fig. 4A). Inhibition of the mitochondrial ATP synthase with oligomycin resulted as expected in decreased cellular ATP levels (Fig. 4C) but had little effect on mitochondrial polarization (14% reduction in polarized conjugates; *P* > 0.5) (Fig. 4A), suggesting that *de novo* ATP synthesis is not required for rapid organelle polarization to the HIV-1 VS.

Increasing levels of cytosolic calcium following release from internal stores trigger extracellular calcium influx via plasma membrane-associated calcium channels, an important step in sustained T cell signaling at the IS (44). In light of our data suggesting a link between cytosolic calcium and T cell polarization, we next investigated whether extracellular calcium influx was necessary for contact-induced polarization. Under conditions where extracellular calcium was chelated with EGTA (10 mM) and intracellular calcium buffered with BAPTA-AM (10 μM), we found that following contact with target cells, HIV-1-infected T cells completely failed to polarize mitochondria and Gag to the contact zone (Fig. 4D and E).

### Analysis of calcium flux at T cell contacts.

**FIG 5:**
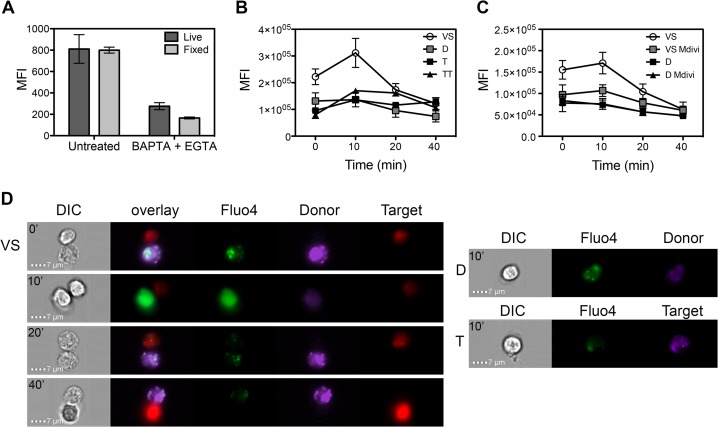
Cell-cell contact is associated with dynamic calcium flux in HIV-1-infected T cells. (A) Fluorescent signal of the calcium indicator Fluo4 is retained after fixation with formaldehyde and is reduced in the presence of the calcium chelators BAPTA and EGTA (10 μM and 10 mM, respectively). (B) An ImageStream-based assay was used to measure intracellular calcium. The mean fluorescent intensity (MFI) of the calcium indicator Fluo4 was measured in infected cells (primary CD4 T cells; Violet) engaged in a VS with an uninfected target cell (primary CD4 T cells; FarRed) (VS, donor plus target) or in single cells alone (D, single donor cell; T, single target) or between two target cells (TT, target plus target). (C) As in panel B but in the presence of Mdivi (no drug versus Mdivi, *P* < 0.005). Quantification of Fluo4 MFI in panels B and C was performed on samples with *n* = 3,501 ± 456 (SEM) events after gating, per experiment (data are from three experiments). (D) Left panel, representative images of contacts formed between HIV-1-infected cells (CellTrace Violet and Fluo4) and targets (red) (ImageStream, 60× objective). Note time-dependent changes in green fluorescence (Fluo4) in HIV-1-infected cells. Right panel, representative images of single HIV-1-infected cells (D) and single target cells (T) loaded with Fluo4 indicator.

Our data with 2-APB and the calcium-chelating agents suggest that intracellular calcium concentrations may be dynamic during VS formation, similar to those of the IS. We therefore set out to image calcium in infected cells during cell-cell contact using ImageStream (Amnis, ED Millipore) and the Ca^2+^ probe Fluo4. Fluo4 has been extensively used as a calcium indicator in live cells (27, 44, 45), and we confirmed that it is retained after fixation in formaldehyde (Fig. 5A). HIV-1-infected primary T cells were loaded with Fluo4 and CellTrace Violet and mixed with uninfected autologous target T cells that were prelabeled with CellTrace FarRed. Cells were allowed to interact for 0, 10, 20, and 40 min, after which they were fixed in formaldehyde and analyzed using ImageStream. Acquisition gating was performed for single cells and doublets using nonfluorescent parameters (scatter, area, and aspect ratio) and then further enriched for doublets formed between one infected (Violet) and one uninfected (FarRed) target cell (see Materials and Methods). The mean fluorescent intensity of the calcium indicator Fluo4 for the resulting populations (average number of cells analyzed per sample, *n* = 3,501 ± 456) was then quantified. Figure 5B shows that HIV-1-infected T cells engaged in a contact with uninfected target cells displayed a transient increase in Fluo4 (calcium) signal after 10 min of coculture and that this progressively decreased to basal levels in the following 30 min (time points, 20 and 40 min) (Fig. 5B). That the calcium signal was lower at 20 and 40 min than at 0 min may reflect the inherent lag time taken to fix cells at *t* = 0 min and the rapid nature of dynamic calcium flux that can occur within minutes at immune cell contacts (23, 46). In contrast, single infected cells (donors) and single uninfected cells (targets), when not engaged in a contact, showed a stable Fluo4 signal over the time course (Fig. 5B). Representative images are shown in Fig. 5D. In order to assess the contribution of infection to the calcium signal, we analyzed doublets formed by two uninfected cells (target-target [TT]). A small, nonsignificant increase in calcium concentration was observed during the course of the experiment (Fig. 5B). Overall, these data show that HIV-1-infected cells engaged in a synapse undergo a transient contact-dependent increase in cytoplasmic calcium concentration that is not seen in uninfected target cells or in infected cells not engaged in a contact.

Mitochondria are known to play a key role in buffering cytosolic calcium at immune cell contacts for sustained signaling at immunological synapses (23, 25, 27). Therefore, to investigate the contribution of mitochondria to the transient calcium increase that we observed in infected cells during cell-cell contact, a Fluo4-ImageStream assay was performed using infected primary CD4 T cells pretreated with Mdivi. Figure 5C shows that Mdivi treatment (which inhibits mitochondrial trafficking to the VS [Fig. 3]) blocked the increase in Fluo4 calcium signal in the HIV-1-infected T cell following cell-cell contact (no drug versus Mdivi, *P* < 0.005). These data suggest that unperturbed mitochondrial trafficking may contribute to dynamic calcium flux at the VS.

### Inhibition of VS formation.

**FIG 6:**
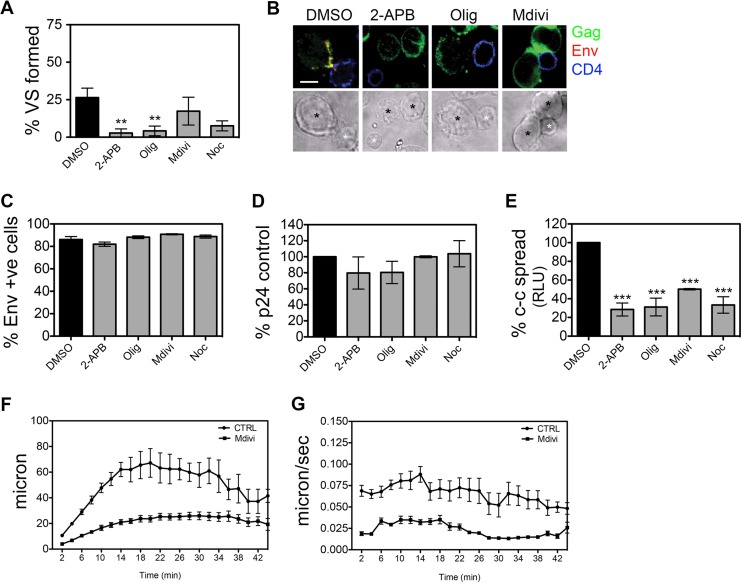
Treatment with inhibitors reduces VS formation and cell-cell spread. (A) VS formation of HIV-1-infected cells treated with 2-APB, Olig, Mdivi, Noc, or DMSO (data from three independent experiments). (B) Representative images from panel A. Green, Gag; red, Env; blue, CD4. (C) Env surface expression of cells treated with inhibitors as in panel A. (D and E) Cell-free virus production from HIV-1-infected cells treated as for panel A was quantified by p24 ELISA (D), and cell-cell (c-c) spread to target T cells (Jurkat 1G5) was quantified by luciferase assay (RLU, relative light units) (E). Data are the means and SEMs from 3 independent experiments. (F and G) Cell motility in the presence of Mdivi or CTRL was assessed by live-cell imaging. HIV-1-infected primary CD4 T cells were treated with Mdivi or DMSO control as described above. Random fields were imaged every 2 min for 46 min, and the distance traveled by the cells (μm) (F) and the speed (μm/s) (G) were measured (*n* = 140 ± 34 cells per treatment per experiment, 2 experiments). For all data: **, *P* < 0.01; ***, *P* < 0.001.

During our analysis of organelle polarization, we noticed that in addition to blocking mitochondrial polarization to the VS, there was a general decrease in the frequency of VS that formed when HIV-1-infected T cells were treated with 2-APB, oligomycin, and Mdivi. To quantify this and determine whether calcium dynamics (2-APB), mitochondrial ATP synthesis (oligomycin), and mitochondrial trafficking (Mdivi) were required for efficient VS formation, synapses were quantified in the presence of the inhibitors. We found that all inhibitors reduced the ability of infected T cells to form VS with uninfected cells (*P* < 0.01) (Fig. 6A and B). In agreement with previous reports, nocodazole also had an inhibitory effect (9). RNAi knockdown of Drp1 also reduced VS formation by 40% (*P* < 0.01; data not shown). Env is necessary for VS formation by engaging CD4 on the target cell, so we investigated if the inhibitor treatment affected Env expression at the plasma membrane. None of the inhibitors decreased surface Env expression as measured by flow cytometry (Fig. 6C). Therefore, it appears that while the presence at the plasma membrane of molecules known to be involved in VS formation (i.e., Env and CD4) is necessary for the synapse to form, they alone are insufficient to establish a stable and productive synapse if mitochondrial trafficking, ATP production, and Ca^2+^ store emptying are impaired.

The observed reduction in VS formation would be expected to impair cell-cell spread. To test this, HIV-1-infected cells were pretreated for 30 min with 2-APB, oligomycin, Mdivi, and nocodazole and cell-cell spread was quantified using target T cells expressing a luciferase reporter gene (31). Figure 6D shows that compared to cells treated with a solvent (DMSO), all inhibitors tested had no effect on cell-free HIV-1 budding but significantly reduced cell-cell transmission of HIV-1 (*P* < 0.001; Fig. 6E).

Having shown that inhibiting mitochondrial trafficking with Mdivi impacted dynamic calcium flux, VS formation, and cell-cell spread, we next considered whether inhibiting mitochondrial movement may also impair T cell motility, which might in turn impact synapse formation (42, 47). HIV-1-infected primary CD4 T cells were loaded with CellTrace Green, incubated with Mdivi or carrier control (DMSO), and imaged every 2 min for 46 min at 37°C with 5% CO_2_. Analysis of the cell tracks (*n* = 140; SEM, ±34 cells per treatment per experiment) (Fig. 6F and G) revealed that Mdivi-treated cells covered shorter distances and at a lower speed compared to the control, suggesting that their migratory ability and, consequently, VS formation potential were impaired. Importantly, since we have observed that Mdivi treatment does not significantly reduce cellular ATP levels (Fig. 3H and I), the observed reduced motility is likely a result of impaired mitochondrial trafficking rather than ATP availability, which would be required to drive energy-dependent movement.

## DISCUSSION

HIV-1 transmission across adhesive T cell-T cell contacts at the VS provides a distinct advantage for viral replication, and numerous studies have attested to the importance of this mode of viral transmission both *in vitro* and *in vivo* (1–5, 7, 8, 11, 12, 19, 20). It is well established that during cell-cell spread the HIV-1-infected CD4 T cell displays a polarized plasma membrane morphology, characterized by the accumulation of viral Env and Gag and cellular proteins at the synaptic interface that serves to focus viral egress toward the target cell (1, 4, 6, 7). Notably, the VS is also associated with striking cytoplasmic polarization within the HIV-1-infected cell toward the contact zone (31); however, the underlying causes and consequences of this for cell-cell spread were unclear. Here, we show that physical contact between HIV-1-infected T cells and uninfected T cells triggers dynamic T cell responses, leading to localized clustering of mitochondria within HIV-1-infected cells proximal to the VS. Additionally, we observed a transient increase in intracellular cytoplasmic calcium concentration in infected cells engaged in a VS that was dependent on unimpaired mitochondrial trafficking. The cytoplasmic compartmentalization and unperturbed mitochondrial function that we observed were required for efficient HIV-1 VS formation. In contrast, equivalent responses were not seen in uninfected T cells. Taken together, we propose a model whereby HIV-1 infection primes T cells for receptor-mediated, calcium-dependent mitochondrial translocation to the contact zone, thus creating a localized environment that supports efficient HIV-1 VS formation and transmission between CD4 T lymphocytes.

*In vivo*, HIV-1 predominantly replicates in CD4 T cells. Lymphoid tissue, which contains densely packed CD4 T cells, is an important site of HIV-1 replication and one that favors contact-dependent transfer (19, 38, 48). The T cell population within lymphoid tissue consists of both nonpolarized T cells and cells prepolarized as a result of prior antigenic stimulation via an APC, migration, or chemokine exposure. The latter display elongated cell morphology, with a clearly defined leading edge and a trailing uropod, and asymmetric cytoplasmic organization, with organelles such as the MTOC and mitochondria concentrated at the uropod (49). In prepolarized T cells, HIV-1 Gag has been shown to localize to uropods where cell-cell contacts and VS formation can occur (3, 50). However, whether polarization could also be specifically triggered by cell-cell contact or if cells can remodel in response to contact and the functional consequences thereof was not known. Here, we find that HIV-1-infected CD4 T cells that display a round morphology and a diffuse network of mitochondria are in fact able to sense and respond to contact with uninfected cells. This leads to a dramatic and rapid remodeling of the cytoplasm that is not accompanied by major cellular morphological changes (i.e., development of leading edge and uropod). Although this cytoplasmic polarization is reminiscent of the immunological synapse between an APC and a T cell, the HIV-1-induced VS is not generated as a result of antigenic stimulation and therefore the signaling and effector pathways might overlap only in part.

It has been shown *in vivo* that HIV-1-infected T cells are highly motile and that migration contributes to spread within the lymphoid tissue (19). Here, using primary CD4 T cells, we show that mitochondria are actively recruited to the VS and that inhibiting mitochondrial trafficking reduces T cell motility. Consequently, VS formation and cell-cell spread are impaired when mitochondrial movement and function are blocked. It will clearly now be worthwhile to define the critical signaling pathways that regulate VS formation and cell-cell spread. This will not only provide mechanistic insight but may also reveal novel therapeutic targets to specifically limit this mode of HIV-1 dissemination.

## Supplementary Material

Supplemental material
