# Supplementary material for: Contact-Induced Mitochondrial Polarization Supports HIV-1 Virological Synapse Formation
Source: J Virol. 2014 Dec 16;89(1):14–24. doi: 10.1128/JVI.02425-14 (PMC4301097; doi:10.1128/JVI.02425-14)
Supplement: Supplemental material [file supp_89_1_14__index.html]

Contact-Induced Mitochondrial Polarization Supports HIV-1 Virological Synapse Formation — Supplemental material 

# Contact-Induced Mitochondrial Polarization Supports HIV-1 Virological Synapse Formation

## Supplemental material

**Files in this Data Supplement:**

- Supplemental file 1 -

  Legends to Movies S1 to S8

  PDF, 61K
- Supplemental file 2 -

  Movie S1 (Volume-rendered three-dimensional reconstruction of an HIV-1-infected CD4 T cell engaged with an uninfected target CD4 T cell.)

  MOV, 149K
- Supplemental file 3 -

  Movie S2 (Live-cell imaging of mitochondrion recruitment to the contact zone formed between an HIV-1-infected Jurkat CD4 T cell and an uninfected target T cell [duration, 48 minutes].)

  MOV, 584K
- Supplemental file 4 -

  Movie S3 (Live-cell imaging of mitochondrion recruitment to the contact zone formed between an HIV-1-infected Jurkat CD4 T cell and an uninfected target T cell [as described for Movie S2; duration, 34 minutes].)

  MOV, 226K
- Supplemental file 5 -

  Movie S4 (Live-cell imaging of mitochondrion recruitment as described for Movie S2 except that primary CD4 T cells were infected with HIV-1 Gag-GFP reporter virus and mixed with autologous CD4 T cells [duration, 38 minutes].)

  MOV, 122K
- Supplemental file 6 -

  Movie S5 (Live-cell imaging of mitochondrion recruitment as described for Movie S2 except that primary CD4 T cells were infected with HIV-1 Gag-GFP reporter virus and mixed with autologous CD4 T cells [as described for Movie S4; duration, 28 minutes].)

  MOV, 109K
- Supplemental file 7 -

  Movie S6 (Live-cell imaging showing that transient contacts between HIV-1-infected T cells and target cells fail to induce polarization.)

  MOV, 642K
- Supplemental file 8 -

  Movie S7 (Volume-rendered three-dimensional reconstruction of a polarized HIV-1-infected CD4 T cell treated with DMSO engaged with an uninfected target CD4 T cell.)

  MOV, 163K
- Supplemental file 9 -

  Movie S8 (Volume-rendered three-dimensional reconstruction of a nonpolarized HIV-1-infected CD4 T cell pretreated with 50 µM Mdivi engaged with an uninfected target CD4 T cell.)

  MOV, 87K
