# Supplementary material for: Contact-Induced Mitochondrial Polarization Supports HIV-1 Virological Synapse Formation
Source: J Virol. 2014 Dec 16;89(1):14–24. doi: 10.1128/JVI.02425-14 (PMC4301097; doi:10.1128/JVI.02425-14)
Supplement: Supplemental material [file JVI.02425-14_zjv999099878so1.pdf]

Supplementary Movie 1. Volume rendered three-dimensional reconstruction of an HIV-1 infected CD4 T cell engaged with an uninfected target CD4 T cell. Mitochondria are red, HIV-1 Gag is green and the uninfected target CD4 T cell is labeled with a cytoplasmic dye (blue). Scale bar is 5 microns. Rotation is around the *x* axis.

Supplementary Movie 2. Live cell imaging of mitochondria recruitment to the contact zone formed between an HIV-1 infected Jurkat CD4 T cell and an uninfected target T cell. Mitochondria are labeled with mitotracker red, HIV-1 infected cells express Gag-GFP. Uninfected target CD4 primary T cells are unlabeled. Images were recorded every 2 minutes using a DeltaVision ELITE Image Restoration Microscope in Real-Time-Z-Sweep mode, in which the Z dimension is acquired continuously and automatically converted to maximum intensity projection. Duration of movie is 48 minutes (played at 1.5 fps) and is representative of n = 20 individual contacts showing recruitment of Gag-GFP and mitochondria to the contact zone.

Supplementary Movie 3. As described for Supplementary Movie 2. Duration of movie is 34 minutes (played at 1.5 fps).

Supplementary Movie 4. As described for Supplementary Movie 2 except primary CD4 T cells were infected with HIV-1 Gag-GFP reporter virus and mixed with autologous CD4 T cells. Duration of movie is 38 minutes (played at 1.5 fps) and is representative of n = 25 individual contacts showing recruitment of Gag-GFP and mitochondria to the contact zone.

Supplementary Movie 5. As described for Supplementary Movie 4. Duration of movie is 28 minutes (played at 1.5 fps).

Supplementary Movie 6. Live cell imaging showing that transient contacts between HIV-1 infected T cells and target cells fail to induce polarization. Duration of movie is 62 minutes (played at 1.5 fps).

Supplementary Movie 7. Volume rendered three-dimensional reconstruction of a polarized HIV-1 infected CD4 T cell (bottom left) treated with DMSO (solvent control) that is engaged with an uninfected target CD4 T cell (top). Mitochondria are red, HIV-1 Gag is green and the cytoplasmic dye labeled target CD4 cell is blue. Rotation is around the *x* axis.

Supplementary Movie 8. Volume rendered three-dimensional reconstruction of a non- polarized HIV-1 infected CD4 T cell (bottom right) pretreated with 50μM Mdivi that is engaged with an uninfected target CD4 T cell (top). Mitochondria are red, HIV-1 Gag is green and the cytoplasmic dye labeled target CD4 cell is blue. Note the elongated mitochondria tubules resulting from inhibition of Drp1 with Mdivi. Rotation is around the *x* axis.
